# The Co-Expression of *GmCML27* and *GmU2AFb* Enhances Tolerance to Alkaline Stress in *Lupinus angustifolius*

**DOI:** 10.3390/plants15142196

**Published:** 2026-07-17

**Authors:** Mengyu Zhou, Yijia Ruan, Hongli Wang, Xiaoyu Wang, Yujing Liu, Xinlei Du, Yishan Fu, Teng Zhang, Jintong Wang, Jie Zhang, Junfeng Zhang, Lei Cao

**Affiliations:** 1 College of Horticulture, Northeast Agricultural University, Harbin 150036, China; zhoumengyu2025@163.com (M.Z.); ryj2504019012@163.com (Y.R.); whl22228888@163.com (H.W.); wxy766696114@163.com (X.W.); yujingliu0000@163.com (Y.L.); duxinlei0101@163.com (X.D.); fys01305611@163.com (Y.F.); zhangteng0012@163.com (T.Z.); wjt1482992725@163.com (J.W.); jiezhang04660421@163.com (J.Z.); 2 School of Geography and Tourism, Harbin University, Harbin 150030, China; jfzhang@hrbu.edu.cn

**Keywords:** alkali stress, *Lupinus angustifolius*, *GmCML27*, *GmU2AFb*, oxidative stress, stress-responsive genes

## Abstract

Soil salinization severely constrains plant growth and agricultural productivity. Identifying key genes conferring alkali tolerance and elucidating their regulatory mechanisms is of great significance for breeding salt–alkali-tolerant leguminous crops. In this study, we investigated the soybean genes *GmCML27* and its potential interacting partner GmU2AFb, and explored their functions in response to alkali stress in narrow-leaf lupine (*Lupinus angustifolius*) through bioinformatics analyses. Protein interaction prediction and experimental validation further supported a potential association between GmCML27 and GmU2AFb. Using an *Agrobacterium rhizogenes* (*A. rhizogenes*)-mediated hairy root transformation system, composite plants carrying empty vector (CK), *GmCML27*-overexpression construct (GC), *GmU2AFb*-overexpression construct (GU), or both *GmCML27* and *GmU2AFb* overexpression constructs (GCU) were generated and evaluated under NaHCO_3_-induced alkali stress. The results showed that all transformed composite plants exhibited enhanced alkali tolerance, with the GCU plants displaying the strongest phenotype. Compared with CK plants, transformed composite plants maintained higher catalase (CAT) activity, proline (Pro) content, and root vitality, while accumulating lower levels of reactive oxygen species (ROS) and malondialdehyde (MDA), and retaining more intact root architecture. Furthermore, the expression levels of alkali tolerance-related genes, including *LaMYB39*, *LaSOS1*, *LaNHX6*, *LaKIN*, *LaDnaJ1*, and *LaP5CS*, were significantly upregulated in transgenic lines. Collectively, our findings demonstrate that both *GmCML27* and *GmU2AFb* positively regulate alkali tolerance in lupine, and their co-expression confers enhanced alkali tolerance compared with single-gene overexpression. These two genes may jointly contribute to alkali stress adaptation through regulation of antioxidant capacity and stress-responsive pathways. This study highlights the potential roles of *GmCML27* and *GmU2AFb* in lupine responses to alkali stress and provides candidate gene resources for future improvement of lupine alkali tolerance.

## 1. Introduction

Soil salinization has become one of the most important abiotic stress factors limiting agricultural production and plant growth, and it continues to exert a persistent impact on the stability of global agroecosystems [1]. Alkali stress is mainly caused by the accumulation of alkaline salts such as NaHCO_3_ and Na_2_CO_3_, and is commonly found in saline–alkaline lands, arid and semi-arid regions, and secondarily salinized soils [2]. Alkali stress not only induces Na^+^ toxicity and osmotic imbalance but also further disrupts cellular ion homeostasis and membrane integrity due to the high-pH environment, while significantly reducing root vitality and photosynthetic efficiency [3]. Roots are the primary organs that first perceive changes in the soil environment; their growth status directly affects the plant’s ability to absorb water and nutrients. Therefore, root damage is generally considered an important indicator of plant sensitivity to alkali stress [4]. In addition, alkali stress triggers massive accumulation of ROS in plants, leading to membrane lipid peroxidation, protein damage, and disruption of cellular structures [5,6,7]. Previous studies have shown that sustained oxidative stress is often accompanied by impaired ion homeostasis and disruption of normal metabolic processes, ultimately inhibiting plant growth and development and weakening stress adaptability [8,9,10]. Consequently, enhancing antioxidant defense capacity, maintaining ROS homeostasis, and alleviating oxidative damage in roots are considered important physiological bases for plant adaptation to alkali stress.

Lupine is an important leguminous plant with ornamental, forage, and ecological application values. It possesses high plant protein content and strong nitrogen-fixing ability, and thus holds broad application prospects in feed development, green manure utilization, horticultural ornamentation, and ecological restoration [11,12,13]. With the increasing demand for plant protein resource exploitation and eco-agricultural development, the utilization value of lupine has attracted growing attention [14]. However, lupine grows optimally in acidic or neutral soils and exhibits relatively weak adaptability to alkaline soils [15]. Alkali stress significantly inhibits seed germination, root elongation, biomass accumulation, and normal plant development in lupine, while inducing excessive ROS accumulation and membrane lipid peroxidation [16,17]. Previous studies have shown that narrow-leaf lupine displays alkali sensitivity due to the absence of *HD-Zip* transcription factors, whereas heterologous expression of the wild soybean *HD-Zip* transcription factor *GsHZ4* significantly enhances plant alkali tolerance [17]. Further studies revealed that the expansion gene *GsEXPA8* can respond to abscisic acid and auxin signals, improving alkali tolerance in soybean and lupine by regulating root development and stress-responsive gene expression, and its homolog *LaEXPA8* is also involved in the alkali stress response of lupine [16]. In addition, *GsHZ4* can target RNA splicing factors, heat shock proteins, and various signaling pathway genes, simultaneously participating in alkali stress responses [18]. These findings suggest that, in addition to conventional transcriptional regulation, RNA processing and post-transcriptional regulation may also play important roles in plant alkali tolerance. Current research on lupine has mainly focused on germplasm evaluation, nutritional quality, population genetic evolution, and responses to abiotic stresses such as drought and high temperature [19,20,21,22,23]. For example, studies have examined the effects of water stress on soluble sugar accumulation [24] and the regulatory effects of high-temperature stress on yield formation in lupine [25]. However, research on the physiological mechanisms of alkali tolerance, mining of alkali-tolerance genes, and creation of alkali-tolerant germplasm in lupine remains relatively limited [26]. Therefore, mining key functional genes from stress-tolerant plants and improving lupine alkali tolerance through genetic engineering are of great significance for expanding its cultivation range and improving the utilization efficiency of saline–alkaline lands.

Ca^2+^ serves as a critical second messenger in plant cells and plays a key regulatory role in sensing and responding to external stress stimuli [27]. When plants are exposed to salt, alkali, drought, and low-temperature stresses, intracellular Ca^2+^ concentrations change rapidly and activate downstream stress response pathways [28,29,30,31]. Calmodulin-like proteins (CMLs) are a family of important Ca^2+^ sensor proteins unique to plants [32], typically characterized by the presence of conserved EF-hand motifs that can bind Ca^2+^ and participate in stress-related signal transduction [33]. Studies have shown that CML family members are widely involved in plant responses to salt, oxidative, and drought stresses [34,35,36]. For instance, Arabidopsis *AtCML9* participates in salt stress and ABA response regulation [37]. Additionally, several CML members have been found to be involved in stress responses in rice, soybean, and other plants [38,39]. Wild soybean (*Glycine soja*), as the ancestor of cultivated soybean, possesses strong environmental adaptability and abundant stress-tolerance gene resources [40]. Previous studies have shown that *GsCML27* from wild soybean is involved in the plant response to alkali stress [41]. NCBI sequence alignment results show that *GmCML27* shares 100% coding sequence identity with *GsCML27*, suggesting that they may have conserved functions in stress responses. Although CML family members have been identified in lupine, their functional roles in alkali stress responses remain largely unknown. Therefore, GmCML27, rather than a lupine endogenous CML member, was selected in this study for heterologous functional evaluation based on its previously characterized alkali-responsive function. Meanwhile, previous studies have shown that the RNA splicing factor *GsU2AFb* is positively regulated by *GsHZ4* and significantly upregulated under alkali stress [18]; *GsU2AFb* shares 99.68% sequence identity with *GmU2AFb*, suggesting conserved functions between these homologous proteins. Importantly, previous interaction screening in our group identified *GmU2AFb* as a candidate interacting partner of *GmCML27*, and subsequent Y2H, BiFC, and LCI assays supported their physical interaction. AlphaFold3 modeling provided additional structural evidence for their potential association. However, whether *GmCML27* and *GmU2AFb* are functionally involved in plant alkali stress responses and whether their co-expression contributes to enhanced alkali tolerance in lupine remain unclear.

Based on the above background, this study selected *GmCML27*, which has stronger evidence for involvement in alkali stress responses, and its potential interacting partner *GmU2AFb* as research subjects. We constructed single-gene overexpression and co-transformation materials in narrow-leaf lupine and simulated alkali stress conditions to systematically evaluate their roles in lupine alkali tolerance. Through phenotypic observation, root scanning, physiological index measurement, and expression analysis of related genes, we compared the differential responses of various transgenic lines to alkali stress, with a particular focus on their effects on the antioxidant defense system, ROS homeostasis maintenance, and osmotic adjustment capacity. This study aims to investigate the roles of *GmCML27* and *GmU2AFb* in the alkali stress response of lupine and evaluate whether their combined expression provides additional benefits for alkali tolerance. These findings provide new insights into the functional roles of CML family genes and U2AF-related regulatory components in plant stress adaptation.

## 2. Results

### 2.1. Phylogenetic Analysis of GmCML27

**Figure 1:**
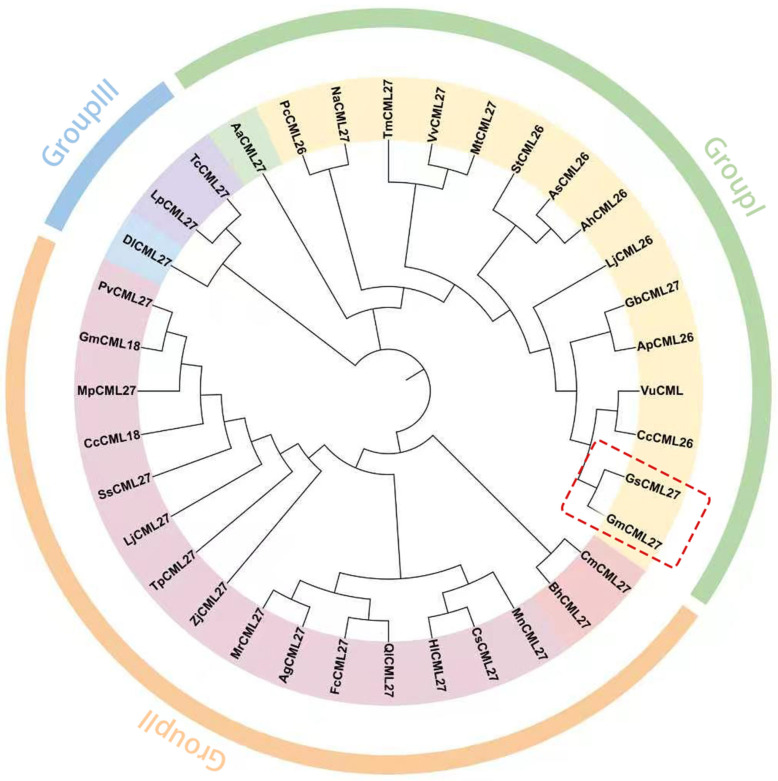
Phylogenetic tree of CML family proteins from different species. The Neighbor-Joining (NJ) phylogenetic tree was constructed using MEGA 11 software. The reliability of the tree was evaluated by 1000 bootstrap replicates. Based on protein sequence similarities and phylogenetic relationships, all CML members were divided into three major clades (Groups I–III). The red dashed box highlights the clade containing the target gene and related genes.

To elucidate the phylogenetic position of *GmCML27* within the CML family, a phylogenetic tree of CML proteins from different species was constructed (Figure 1). The results showed that all CML proteins could be divided into three major clades (Groups I–III), containing 16, 17, and 3 members, respectively, indicating evolutionary divergence within this family. Distinct phylogenetic differentiation was observed among different clades, whereas members within the same clade exhibited close clustering relationships. *GmCML27* clustered most closely with *GsCML27* and grouped in the same clade with CML proteins from several leguminous species, suggesting a high degree of evolutionary conservation within legumes. Notably, no lupine CML member was found to cluster with *GmCML27* in the phylogenetic analysis.

### 2.2. Conserved Motif and Domain Analysis of GmCML27

**Figure 2:**
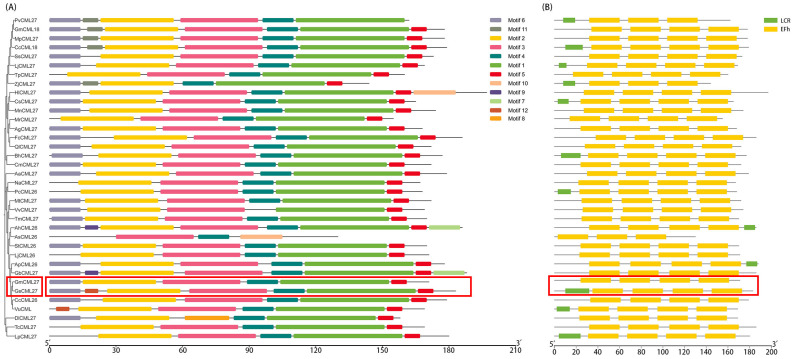
Phylogenetic analysis and conserved domain analysis of CML family proteins. (**A**) A Neighbor-Joining phylogenetic tree was constructed using MEGA 11 software with 1000 bootstrap replicates. The conserved motifs of *GmCML27* protein were predicted using the online tool MEME. (**B**) Domain analysis of *GmCML27* protein was performed using the NCBI Conserved Domain Database and Pfam database. These analyses were integrated with the phylogenetic tree using TBtools-II v2.210. The red box highlights the target gene and related genes in the conserved motif and domain analysis.

To characterize the conserved features of CML family proteins, the conserved motifs were predicted using the MEME program. Furthermore, domain prediction of the *GmCML27* protein was performed using the NCBI Conserved Domain Database (CDD) and the Pfam database (Figure 2A,B). A total of 12 conserved motifs (Motifs 1–12) were identified. Among them, Motifs 2, 3, 4, 5, 6, and 7 were widely present in most CML proteins, whereas some motifs were found only in specific members. Overall, CML proteins within the same phylogenetic clade generally shared similar motif compositions and arrangement patterns. *GmCML27* contained several core conserved motifs of the family, and its motif composition was essentially identical to that of *GsCML27*, further supporting the high structural conservation between the two proteins. Moreover, *GmCML27* contained only the typical EF-hand calcium-binding domain, with no other functional domains detected. Concurrently, most CML family members possessed EF-hand-related domains, indicating that this domain is an important conserved feature of the CML family.

### 2.3. Chromosomal Localization, Synteny Relationships, and Promoter Cis-Acting Element Analysis of the Lupine CML Family

**Figure 3:**
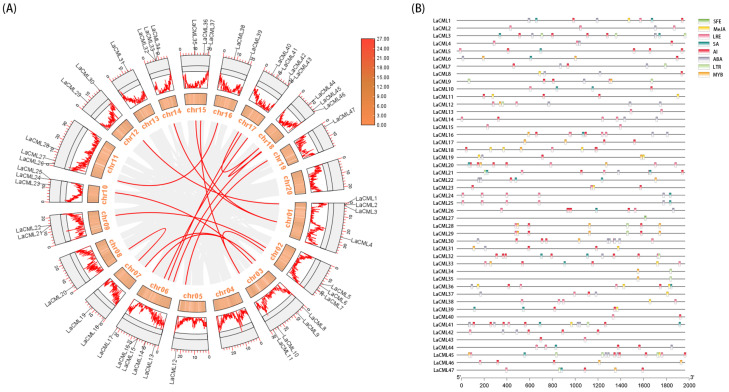
Chromosomal localization, promoter cis-acting elements, and synteny analysis of CML family genes in lupine. (**A**) Chromosomes are represented as circles using TBtools-II v2.210 software. Red lines connect duplicated gene pairs. (**B**) Distribution of cis-acting elements in the promoters of lupine CML family genes. The 2000 bp sequences upstream of the start codon of each lupine CML member were extracted, and cis-acting elements were predicted using the PlantCARE online tool. The predicted elements were visualized as color-coded and numbered boxes via TBtools.

Given that *GmCML27* belongs to the CML family, we further analyzed the chromosomal distribution, synteny relationships, and promoter features of the CML family in lupine (Figure 3A). Chromosomal localization results showed that lupine CML family genes are distributed across 18 chromosomes, exhibiting a non-uniform distribution pattern. Some chromosomes harbored multiple lupine CML members, while others contained only 1–2 members, indicating a clear uneven distribution of the CML family in the lupine genome. Synteny analysis revealed that CML family members are located on different chromosomes, and multiple pairs of inter-chromosomal syntenic relationships were detected. Several members exhibited obvious syntenic connections, among which chromosomes chr05, chr06, chr15, and chr16 were involved in relatively more syntenic relationships, whereas only a few syntenic connections were observed on some other chromosomes. Overall, lupine CML members retained abundant homologous relationships in the genome, forming multiple inter-chromosomal syntenic linkages.

Promoter cis-acting element analysis showed that the 2000 bp upstream regions of lupine CML family genes contain various regulatory elements associated with hormone responses and stress adaptation (Figure 3B). These elements mainly include ABA-responsive elements (ABRE), MeJA-responsive elements, low-temperature responsive elements (LTR), anaerobic-inducible elements (ARE), light-responsive elements (LRE), and MYB transcription factor binding sites. The composition and numbers of cis-acting elements varied considerably among different lupine CML promoters, suggesting functional divergence within this family during evolution. Notably, only low-temperature-related elements were detected in the promoter region of *LaCML27*, whereas no obvious alkali-stress-related regulatory elements were found, implying that *LaCML27* may primarily participate in low-temperature adaptation processes.

### 2.4. Prediction of GmCML27 Interacting Proteins

**Figure 4:**
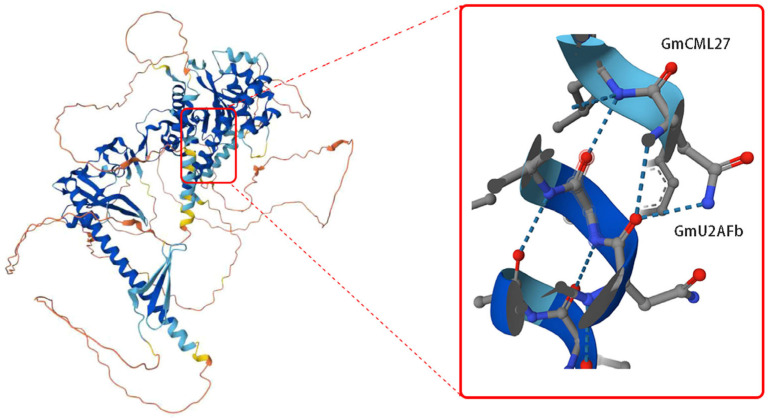
Structural model of the potential protein interaction complex of GmCML27. Three-dimensional structural model of the GmCML27-GmU2AFb complex predicted by AlphaFold3. Dashed lines connect the original structure with the enlarged regions for detailed visualization.

To further investigate the potential interaction between GmCML27 and GmU2AFb, structural prediction and interaction modeling were performed using AlphaFold3. The results showed that GmCML27 and GmU2AFb can form a stable protein complex (Figure 4). The overall structural model indicated good spatial complementarity at the interaction interface between the two proteins. Enlarged views of the interface revealed multiple potential non-covalent interaction sites between them, suggesting that GmCML27 and GmU2AFb may form a stable association. In addition, the predicted aligned error (PAE) analysis showed relatively low prediction errors at the interaction interface, indicating high confidence in the predicted relative orientation of the two proteins (Figure S1). Our previous experimental results have demonstrated an interaction between GmCML27 and GmU2AFb. The structural predictions obtained in this study are consistent with these experimental findings, further supporting the possibility of stable complex formation between the two proteins.

### 2.5. Overexpression of GmCML27 and GmU2AFb Enhances Lupine Tolerance to Alkali Stress

**Figure 5:**
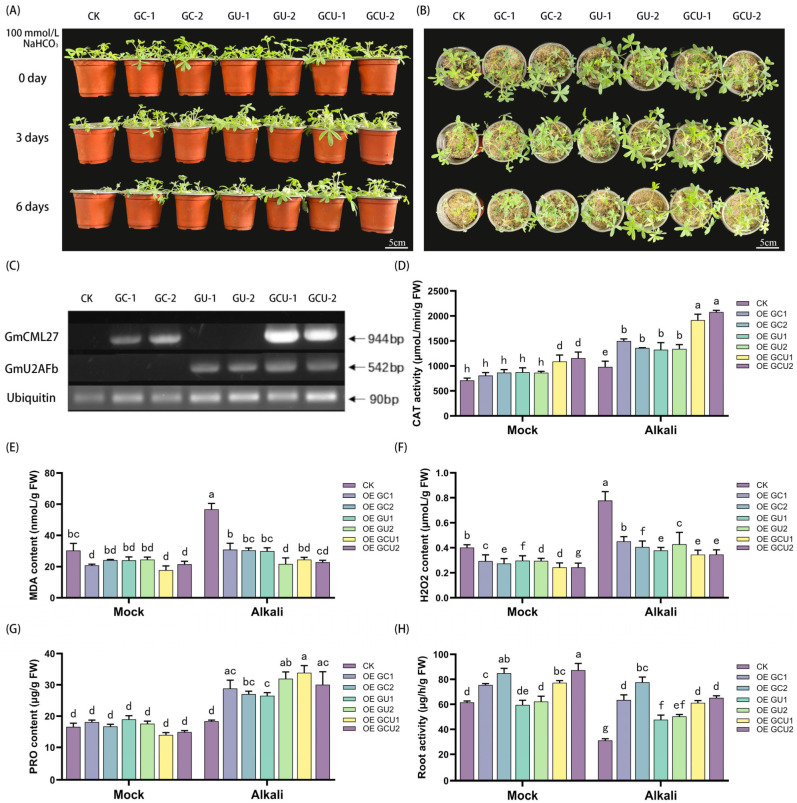
Effects of *GmCML27* and *GmU2AFb* overexpression on alkali tolerance in lupine. (**A**) Phenotypic changes of CK, GC, GU, and GCU plants under 100 mmol·L^−1^ NaHCO_3_ treatment at 0, 3, and 6 days (top view); scale bar = 5 cm. (**B**) Phenotypic changes of the same plants (top view); scale bar = 5 cm. (**C**) RT-PCR confirmation of the presence of introduced *GmCML27* and *GmU2AFb* transcripts in GC, GU, and GCU plants. (**D**) CAT activity. (**E**) MDA content. (**F**) H_2_O_2_ content. (**G**) Proline content. (**H**) Root vitality. Data are presented as mean ± SD (*n* = 3). Statistical analysis was performed using two-way ANOVA. Different lowercase letters indicate significant differences among groups (*p* < 0.05).

Under alkali stress conditions, distinct phenotypic differences were observed among the various lines (Figure 5A,B). Before treatment, all lines exhibited similar growth status. As stress duration progressed, empty pBI121 vector control (CK) plants gradually developed leaf chlorosis and wilting, whereas GC (*GmCML27* overexpression), GU (*GmU2AFb* overexpression), and GCU (*GmCML27-GmU2AFb* co-overexpression) plants displayed relatively milder stress symptoms. After 3 days of treatment, CK plants showed obvious growth inhibition, while transgenic lines maintained good growth. After 6 days of treatment, leaf chlorosis and wilting in CK plants further intensified; GC and GU plants still maintained relatively good growth, whereas GCU plants exhibited the least damage and overall better growth than both single-gene overexpression lines. RT-PCR analysis confirmed the presence of introduced *GmCML27* and *GmU2AFb* transcripts in transformed lupine hairy roots (Figure 5C).

To further analyze the differential responses of various lines to alkali stress, we measured catalase (CAT) activity, malondialdehyde (MDA) content, H_2_O_2_ content, proline (Pro) content, and root vitality. Under normal growth conditions, the physiological indices among all lines showed minimal differences overall (Figure 5D–H). After alkali stress treatment, all lines exhibited varying degrees of physiological responses. CAT activity increased in all lines, with GC, GU, and GCU plants showing higher activities than CK, and GCU plants displaying the highest activity. Meanwhile, MDA and H_2_O_2_ contents increased significantly in all lines, but their accumulation levels in transgenic lines were lower than in CK, with GCU plants showing the lowest levels. Furthermore, Pro content increased markedly under alkali stress in all lines, with transgenic lines accumulating higher levels than CK, and GCU plants showing the highest accumulation. Root vitality was suppressed by alkali stress, but transgenic lines maintained relatively higher levels. Both GC and GU plants exhibited significantly higher root vitality than CK, while GCU plants showed the highest root vitality among all lines. Collectively, the phenotypic and physiological results indicate that overexpression of either *GmCML27* or *GmU2AFb* alleviates the adverse effects of alkali stress on lupine, and co-transformed plants exhibited superior alkali-tolerant phenotypes in terms of plant growth, ROS accumulation, osmotic adjustment capacity, and root vitality.

### 2.6. Overexpression of GmCML27 and GmU2AFb Reduces Alkali Stress-Induced ROS Accumulation in Leaves

**Figure 6:**
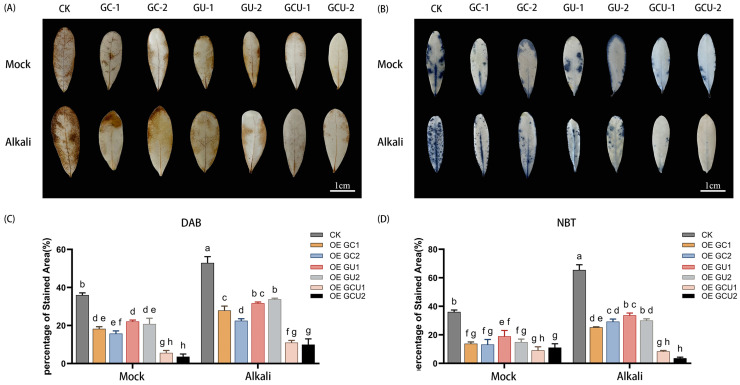
Effects of *GmCML27* and *GmU2AFb* overexpression on alkali stress-induced ROS accumulation in lupine leaves. (**A**) DAB staining of leaves from CK, GC, GU, and GCU plants treated with H_2_O (Mock) or 100 mmol·L^−1^ NaHCO_3_. (**B**) NBT staining of leaves from the same lines under the same treatments. (**C**) Quantitative analysis of the percentage of DAB-stained area. (**D**) Quantitative analysis of the percentage of NBT-stained area. Data in (**C**,**D**) are presented as mean ± SD (*n* = 3). Statistical analysis was performed using two-way ANOVA. Different lowercase letters indicate significant differences among groups (*p* < 0.05). Scale bars = 1 cm.

As roots are the primary organs for sensing and responding to soil alkali stress, their damage is often accompanied by shoot growth inhibition and leaf injury. Therefore, to evaluate the responses of shoot tissues to alkali stress across different lines, we performed diaminobenzidine (DAB) staining and nitroblue tetrazolium (NBT) histochemical staining on leaves, where DAB staining detects H_2_O_2_ accumulation and NBT staining detects O_2_^−^ accumulation. As shown in Figure 6A,B, under normal growth conditions, leaves of all lines exhibited only slight background staining with minor differences among groups. After alkali stress treatment, staining intensity increased markedly in all lines, but significant differences were observed among them. CK leaves displayed extensive DAB brown precipitates and NBT blue patches, with the most pronounced staining; GC and GU leaves showed reduced staining, while GCU leaves exhibited the weakest coloration.

Further quantitative analysis of ROS accumulation levels was performed by measuring the stained areas of DAB and NBT. Under normal conditions, the stained areas were low in all lines. After alkali stress, the stained areas increased significantly in all lines, with the largest increases observed in CK plants. Compared with CK, the stained areas were significantly reduced in GC and GU lines, and even further reduced in GCU lines, which maintained the lowest levels across all treatment groups (Figure 6C,D). Both histochemical staining and quantitative analyses consistently demonstrated that GC, GU, and GCU lines effectively alleviated alkali stress-induced ROS accumulation, with GCU lines showing the most pronounced mitigation effect.

### 2.7. Overexpression of GmCML27 and GmU2AFb Improves Root Growth Under Alkali Stress Conditions

**Figure 7:**
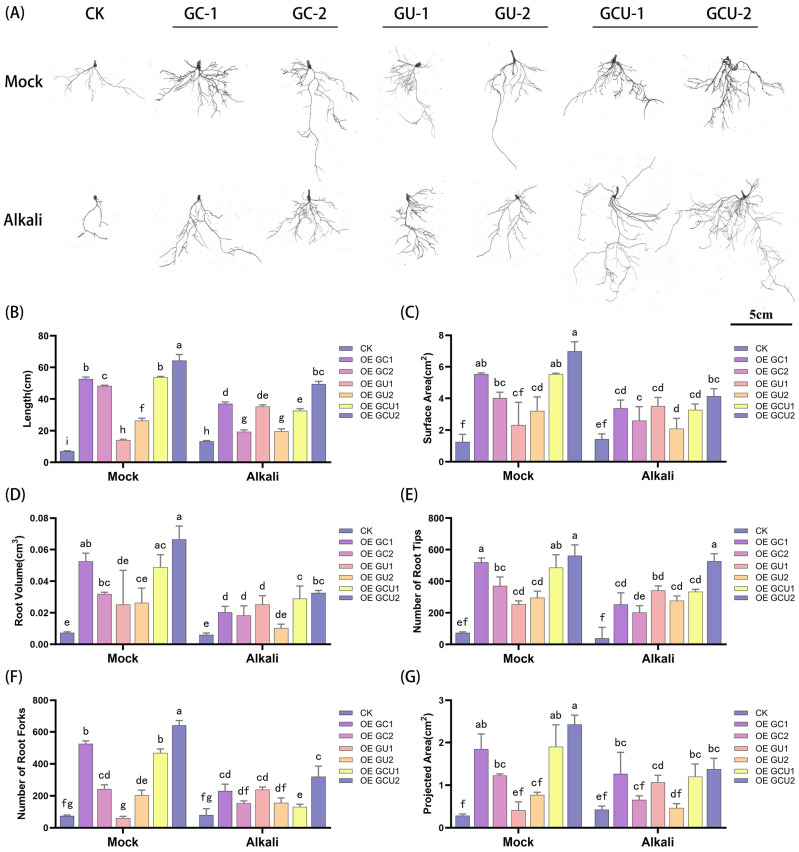
Effects of *GmCML27* and *GmU2AFb* overexpression on root architecture of lupine under alkali stress conditions. (**A**) Root phenotype; scale bar = 5 cm. (**B**) Total root length. (**C**) Root surface area. (**D**) Root volume. (**E**) Number of root tips. (**F**) Number of root forks. (**G**) Projected area. Data in (**B**–**G**) are presented as mean ± SD (*n* = 3). Statistical significance was determined by two-way ANOVA. Different lowercase letters indicate significant differences among groups (*p* < 0.05).

To compare root growth among different lines under alkali stress, the root systems were scanned using the LA-S root analysis system. As shown in Figure 7A, under normal growth conditions, all lines developed relatively intact root architectures, but transgenic plants generally exhibited better root development than CK, with GCU plants showing the most vigorous root systems. After alkali stress treatment, root growth was inhibited to varying degrees across all lines, manifested as reduced root system size and decreased lateral root number. In comparison, CK plants showed the most pronounced inhibition, whereas transgenic lines maintained relatively better root architecture, with GCU plants retaining more lateral roots and greater root mass.

Further measurements were taken for total root length, surface area, root volume, number of root tips, number of root forks, and projected area. The results showed that under normal conditions, transgenic lines exhibited higher root parameters than CK (Figure 7B–G), with total root length, surface area, and root volume all showing varying degrees of increase, while the increases in root tip number and fork number were more pronounced. Notably, GCU lines reached the highest levels in root tip number, fork number, and projected area, indicating a more developed root architecture. After alkali stress treatment, root growth was suppressed in all lines, but the extent of reduction varied significantly among lines. Compared with CK, transgenic lines maintained greater total root length, surface area, and root volume, while also retaining more root tips and lateral root branches. Among them, GCU lines consistently maintained the highest values for most parameters, with particularly notable advantages in root tip number and fork number, demonstrating strong root growth capacity. It is worth noting that under normal conditions, most root parameters of GU lines were higher than those of GC lines; however, under alkali stress, the differences between them diminished. In contrast, GCU lines exhibited the best root growth status under both normal and alkali stress conditions, maintaining the highest levels in most root parameters.

### 2.8. Overexpression of GmCML27 and GmU2AFb Promotes the Expression of Alkali Stress-Responsive Genes

**Figure 8:**
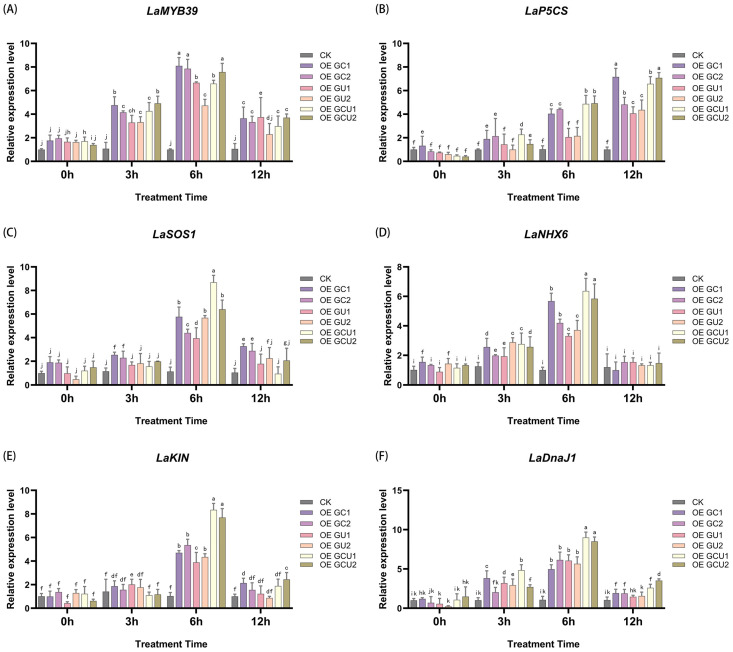
Relative expression levels of alkali stress-responsive genes in CK, GC, GU, and GCU lupine plants at 0, 3, 6, and 12 h after NaHCO_3_ stress treatment. (**A**) *LaMYB39*; (**B**) *LaP5CS*; (**C**) *LaSOS1*; (**D**) *LaNHX6*; (**E**) *LaKIN*; (**F**) *LaDnaJ1*. Data are presented as mean ± SD (*n* = 3). Statistical analysis was performed using two-way ANOVA. Different lowercase letters indicate significant differences among treatments (*p* < 0.05).

To investigate the effects of *GmCML27* and *GmU2AFb* overexpression on the expression of endogenous alkali stress-responsive genes in lupine, q-PCR was employed to detect the expression changes of relevant alkali-tolerance genes at different treatment time points, aiming to characterize the molecular responses of different lines to alkali stress. Based on previous studies, we selected *LaMYB39*, *LaP5CS*, *LaSOS1*, *LaNHX6*, *LaKIN*, and *LaDnaJ1* for analysis, all of which are known to be involved in ion homeostasis maintenance, osmotic regulation, root growth, and stress adaptation. As shown in Figure 8, all of these genes were induced to varying degrees after alkali stress treatment, although their expression patterns differed.

*LaMYB39* expression in GC, GU, and GCU lines increased initially and then decreased, peaking at 6 h. At this time point, expression levels in all transgenic lines were significantly higher than in CK, with GCU lines showing the highest levels (Figure 8A). *LaP5CS* expression in GC, GU, and GCU lines continuously increased with treatment duration, reaching the highest levels at 12 h (Figure 8B). At 3 h and 6 h of alkali stress, expression levels in GC and GCU lines were significantly higher than in CK; at 12 h, all transgenic lines maintained relatively high expression levels, all significantly higher than CK.

*LaSOS1* expression increased initially and then decreased after NaHCO_3_ treatment, peaking at 6 h (Figure 8C). At 3 h, expression in GC lines was significantly higher than in CK; at 6 h, expression in GC, GU, and GCU lines was significantly higher than in CK; at 12 h, GC and GCU lines still maintained relatively high expression levels. *LaNHX6* also showed an initial increase followed by a decrease, peaking at 6 h (Figure 8D). At 3 h and 6 h, expression levels in GC, GU, and GCU lines were significantly higher than in CK, with GCU lines showing the greatest upregulation.

*LaKIN* was markedly induced in all transgenic lines, with expression peaking at 6 h (Figure 8E). At the peak time point, expression levels in all transgenic lines were significantly higher than in CK, with GCU lines showing the highest levels. *LaDnaJ1* was also significantly induced under alkali stress, reaching its highest expression level at 6 h (Figure 8F). At this time point, expression levels in GC, GU, and GCU lines were all significantly higher than in CK, with GCU lines showing the highest levels.

Overall, *LaMYB39*, *LaP5CS*, *LaSOS1*, *LaNHX6*, *LaKIN*, and *LaDnaJ1* were all induced by alkali stress, and their expression levels in transgenic lines were generally higher than those in CK. Compared with single gene overexpression lines, GCU lines exhibited higher expression levels at most time points for the majority of genes, indicating that co-expression of *GmCML27* and *GmU2AFb* more effectively promotes the expression of alkali stress-responsive genes.

## 3. Discussion

### 3.1. Evolutionary Conservation of GmCML27 and Potential Regulatory Features of the Lupine CML Family

CML proteins are important sensors in plant Ca^2+^ signaling networks that perceive changes in intracellular Ca^2+^ concentrations through EF-hand domains and transduce Ca^2+^ signals into downstream physiological responses. Previous studies have shown that the CML family is widely involved in plant adaptation to salt, drought, low-temperature, and pathogen stresses [37,42,43]. Our phylogenetic analysis revealed that *GmCML27* clustered most closely with *GsCML27*, and the two proteins shared highly consistent sequence features. Additionally, GmCML27 contains a typical EF-hand calcium-binding domain, indicating high evolutionary conservation in soybean and wild soybean. Previous studies have confirmed that *GsCML27* is involved in the plant response to alkali stress [41], leading us to speculate that *GmCML27* may retain similar stress-responsive functions and play an important role in plant alkali tolerance.

Gene family expansion is generally considered an important evolutionary basis for plant adaptation to complex environments. Synteny analysis showed that lupine CML members are widely distributed across different chromosomes, forming multiple inter-chromosomal syntenic blocks, with a local gene cluster formed on chr11. Similar phenomena have been reported for CML families in *Arabidopsis*, *rice*, and *soybean*, suggesting that gene duplication events may have contributed to the expansion and retention of the CML family [44]. Gene duplication not only increased the number of family members but also provided a genetic basis for subsequent functional divergence. Therefore, the structural and expression differences among lupine CML members may reflect their adaptive specialization to different environmental signals. Promoter analysis further revealed potential regulatory features of the lupine CML family. ABRE, MeJA-responsive elements, and MYB binding sites were widely present in the promoter regions of most lupine CML members, suggesting that they may be coordinately regulated by both hormonal and stress signals. Previous studies have shown extensive crosstalk among ABA signaling, Ca^2+^ signaling, and ROS signaling in plant stress adaptation [45]. Thus, lupine CML members may serve as important nodal connectors in multiple signaling pathways, playing regulatory roles in plant stress responses.

### 3.2. Overexpression of GmCML27 and GmU2AFb Alleviates Oxidative Damage and Improves Root Growth Under Alkali Stress

In addition to ionic toxicity and osmotic stress, alkali stress also causes nutrient imbalance and metabolic disruption due to high pH, leading to severe damage to plants [46]. Under NaHCO_3_ stress, overexpression of either *GmCML27* or *GmU2AFb* enhanced alkali tolerance in lupine. Notably, GCU plants exhibited stronger tolerance than single-gene overexpression plants, suggesting that the combined expression of *GmCML27* and *GmU2AFb* may provide additional benefits for alkali stress adaptation. Oxidative stress is a major manifestation of alkali-induced damage; NaHCO_3_ treatment induces ROS accumulation and leads to membrane lipid peroxidation [47]. In this study, CK plants exhibited higher ROS and MDA accumulation under alkali stress, indicating more severe oxidative damage, whereas transgenic lines maintained lower ROS and MDA levels accompanied by increased CAT activity. Meanwhile, transgenic lines accumulated more Pro. As a small molecule with both osmotic regulatory and antioxidant functions, Pro can participate in maintaining cellular osmotic balance and protecting cellular structures [48]. Similar phenomena have been observed in studies of stress-responsive genes such as *AtCML37* and *ShCML44*, indicating that enhancing antioxidant capacity is an important approach to improving plant stress tolerance [42,43]. Therefore, we speculate that *GmCML27* and *GmU2AFb* may alleviate alkali-induced oxidative damage by promoting Pro accumulation, enhancing ROS scavenging efficiency, and maintaining cellular homeostasis.

Roots serve as the first barrier for plants to sense and respond to soil salinity and alkalinity. Previous studies have indicated that root length, root tip number, and fork number directly affect plant capacity for water and nutrient acquisition [49]. In this study, alkali stress significantly inhibited root growth in CK plants, whereas transgenic lines maintained better root development, with GCU plants performing best. We speculate that *GmCML27* and *GmU2AFb* may enhance plant adaptation to alkali stress by promoting root development and functional maintenance. Additionally, some root traits of GU lines showed slightly higher values under mild alkali stress than under mock conditions, which may be associated with adaptive plasticity of root systems under moderate stress, allowing plants to maintain or enhance root growth and resource acquisition capacity. The distinct response patterns among independent GU lines may be related to variations among transformation events, and the underlying mechanisms require further investigation.

### 3.3. GmCML27 and GmU2AFb May Participate in Alkali Tolerance Through Multiple Regulatory Processes

Ca^2+^ signaling is one of the earliest signals activated after plants perceive stress stimuli. Salt and alkali stresses rapidly induce elevation of cytosolic Ca^2+^ concentrations, which are recognized by Ca^2+^ sensors such as CMLs and transduced to downstream response networks [45]. As a typical CML family member, *GmCML27* may participate in stress-responsive signaling processes. In this study, *LaMYB39*, *LaP5CS*, *LaSOS1*, and *LaNHX6* exhibited higher expression levels in transgenic lines, suggesting that *GmCML27* may contribute to the activation of alkali stress-responsive pathways. However, whether these transcriptional changes directly affect ion transport or cellular ion homeostasis requires further investigation. Meanwhile, *GmU2AFb* belongs to the RNA splicing factor family. Accumulating evidence indicates that alternative splicing is a key regulatory mechanism in plant stress responses, enabling rapid adaptation to environmental changes by altering transcript structure and expression efficiency [50,51]. As important components of the pre-mRNA splicing machinery, U2AF proteins have been implicated in plant development and stress responses. However, the involvement of U2AF family members in plant alkali stress responses remains largely unexplored. In this study, overexpression of *GmU2AFb* significantly enhanced alkali tolerance in lupine, suggesting that *GmU2AFb* may act as a positive regulator of alkali stress adaptation. although its downstream regulatory mechanisms require further investigation. However, whether *GmU2AFb* mediates alkali tolerance through RNA splicing regulation or other downstream pathways remains to be elucidated.

Notably, *GmU2AFb* was identified as a candidate interacting protein of *GmCML27* based on preliminary screening and prediction analyses. AlphaFold3 structural prediction further provided additional support for a potential association between the two proteins. However, these results do not directly establish a functional connection between Ca^2+^ signaling and RNA splicing regulation. In this study, co-transformed plants exhibited improved phenotypic performance, physiological characteristics, root development, and expression of stress-responsive genes compared with single-gene overexpression plants, suggesting that *GmCML27* and *GmU2AFb* may have combined effects in enhancing alkali tolerance. However, the current evidence is mainly based on phenotypic, physiological, and transcriptional analyses. Further studies integrating Ca^2+^ dynamic analysis, transcriptomic analysis, and alternative splicing profiling are required to elucidate the detailed molecular mechanisms underlying their potential regulatory relationship. It should also be noted that the current study was performed using an *A. rhizogenes*-mediated hairy root transformation system, which is suitable for rapid functional assessment but cannot fully represent the performance of stable transgenic plants or field conditions. Further validation using stable transformation materials and long-term alkali stress evaluations will be necessary to assess the practical application potential of *GmCML27* and *GmU2AFb*.

## 4. Materials and Methods

### 4.1. Plant Material and Culture Conditions

This experiment was conducted at the Horticultural Experiment Center of Northeast Agricultural University, located at 45°44′28″ N, 126°43′16″ E. Lupine seeds were purchased from “Huayouxiu” commercial seedling supplier [16]. To generate overexpression constructs, the coding sequences (CDS) of *GmCML27* and *GmU2AFb* were amplified using gene-specific primers containing homologous recombination sequences at both ends. The binary expression vector pBI121, which contains a CaMV 35S promoter-driven expression cassette, a NOS terminator, and the nptII selectable marker, was used as the expression backbone. The pBI121 vector was linearized by double digestion with BamH I and Sac I, and the amplified CDS fragments were inserted into the linearized vector through homologous recombination [16]. The recombinant plasmids pBI121-*GmCML27* and pBI121-*GmU2AFb* were introduced into *Escherichia coli* DH5α for amplification and verified by PCR and sequencing. The confirmed recombinant plasmids were subsequently transformed into *Agrobacterium rhizogenes* strain K599 (AC1080; Weidi Biotechnology, Shanghai, China) using the freeze–thaw method. The engineered *A. rhizogenes* strains were cultured in LB liquid medium containing 50 mg/L kanamycin at 28 °C with shaking at 200 rpm until the logarithmic growth phase. The bacterial cultures were collected and resuspended to prepare infection suspensions [16,17]. Lupine composite plants were generated using an *A. rhizogenes*-mediated hairy root transformation system according to previously established protocols [17,52]. Briefly, wounds were generated at the root–stem junction of uniformly grown lupine seedlings, and the wounded sites were inoculated with *A. rhizogenes* K599 cultures carrying the corresponding overexpression constructs. For co-overexpression transformation, *A. rhizogenes* cultures harboring pBI121-*GmCML27* and pBI121-*GmU2AFb* were mixed at an equal volume ratio (1:1) before inoculation, while the total bacterial suspension volume was maintained consistent with that used for single-gene transformation groups.

Following transformation, seedlings were transplanted into pots containing a mixture of nutrient soil and vermiculite (1:1, *v*/*v*) and cultivated under controlled environmental conditions at 22–25 °C with a 16 h light/8 h dark photoperiod. Subsequent experiments were performed when the third pair of palmate compound leaves had fully expanded [17]. Based on the introduced constructs, composite plants with root-specific overexpression of *GmCML27*, *GmU2AFb*, and co-overexpression of both genes were generated. Plants infected with *A. rhizogenes* K599 carrying the empty pBI121 vector were used as empty vector controls. Although non-transformed wild-type plants were not included in this study, empty vector-transformed composite plants provided a consistent transformation background for evaluating the effects of *GmCML27* and *GmU2AFb* overexpression.

### 4.2. RT-PCR Identification of Transgenic Root Lines

Root tissues of CK, GC, GU, and GCU plants were collected, immediately frozen in liquid nitrogen, and stored at −80 °C until use. Total RNA was extracted using the TransZol Up Kit (ET111-01-V2; TransGen Biotech Co., Ltd., Beijing, China). First-strand cDNA was synthesized from 1 μg of total RNA using the SPARKSCRIPT II RT Plus Kit (with gDNA Eraser; AG0304-B, Shandong Sparkjade Biotechnology Co., Ltd., Jinan, China) according to the manufacturer’s instructions. RT-PCR was performed using synthesized cDNA as the template, and the amplification products were detected by agarose gel electrophoresis to identify positive transformed lupine hairy root tissues carrying introduced *GmCML27* and *GmU2AFb* transcripts. Primers used for RT-PCR are listed in Table 1.

### 4.3. Plant Phenotyping Under Alkali Stress Treatment

Plants of empty vector controls and transgenic lines at 50 days of age with uniform growth status were selected as experimental materials. Alkali stress was applied by root irrigation with 100 mmol·L^−1^ NaHCO_3_ solution, with distilled water treatment as the control. Both control and treatment groups were set up with at least three biological replicates. During the treatment period, phenotypic changes were continuously monitored. When CK plants exhibited obvious leaf yellowing and wilting symptoms, physiological measurements were performed for CK and transgenic lines [17]. CK plants under NaHCO_3_ treatment served as the alkali stress control, and the phenotypic responses of GC, GU, and GCU plants under alkali stress were compared.

### 4.4. Physiological Index Measurement Under Alkali Stress

The same alkali stress treatment conditions as described in Section 4.3 were applied. After CK plants in the NaHCO_3_ treatment group exhibited obvious leaf chlorosis and wilting phenotypes, root samples from each treatment group were collected for physiological index measurement. Root tissues (0.1 g) were frozen in liquid nitrogen and stored at −80 °C for later use. CAT activity, MDA content, Pro content, H_2_O_2_ content, and root vitality were determined using commercial assay kits. The kits for CAT (No. G0105W), MDA (No. G0109W), Pro (No. G0111W), H_2_O_2_ (No. G0112W), and root vitality (No. G0124W) were purchased from Suzhou Grace Biotechnology Co., Ltd. (Suzhou, China). All measurements were performed according to the manufacturers’ instructions [16,17].

### 4.5. Diaminobenzidine Staining

DAB staining was used to detect H_2_O_2_ accumulation in plant tissues, reflecting the degree of oxidative stress. CK, GC, GU, and GCU plants at 50 days of age with uniform growth were selected. Alkali stress was applied by root irrigation with 100 mmol·L^−1^ NaHCO_3_ solution, with distilled water treatment as the control. At 3 days after treatment, leaves from each treatment group were collected for DAB staining analysis [17].

DAB staining solution was prepared by dissolving 0.1 g DAB powder (Biotopped, Beijing, China) in 50 mL of distilled water, and the pH was adjusted to 5.7. Leaf samples were immersed in DAB staining solution and subjected to vacuum infiltration for 10 min, followed by staining in the dark for 18 h. After staining, leaves were placed in 95% ethanol and decolorized in a 95 °C water bath until completely transparent, and the staining results were photographed [53]. The percentage of stained leaf area was calculated using ImageJ 1.54p software to evaluate H_2_O_2_ accumulation levels in leaves of different treatment groups.

### 4.6. Nitroblue Tetrazolium Staining

NBT staining was used to detect superoxide anion (O_2_^−^) accumulation in plant tissues, reflecting the degree of oxidative stress. CK, GC, GU, and GCU plants at 50 days of age with uniform growth were selected. Alkali stress was applied by root irrigation with 100 mmol·L^−1^ NaHCO_3_, with distilled water treatment as the control. At 3 days after treatment, leaves from each treatment group were collected for NBT staining analysis [17]. NBT staining solution was prepared by dissolving 0.1 g NBT powder (Biotopped, Beijing, China) in 50 mL phosphate buffer (pH 7.5) and stored in the dark. Leaf samples were immersed in NBT staining solution and stained in the dark for 18 h. After staining, leaves were placed in 95% ethanol and decolorized in a 95 °C water bath until completely transparent, and the staining results were photographed [16]. The percentage of stained leaf area was calculated using ImageJ 1.54p software to quantitatively evaluate O_2_^−^ accumulation levels in leaves of different treatment groups.

### 4.7. Root Scanning

To evaluate the effects of *GmCML27*, *GmU2AFb*, and their co-overexpression on lupine root morphological characteristics under alkali stress, CK, GC, GU, and GCU plants at 25 days of age with uniform growth were selected as experimental materials. Alkali stress was applied with 25 mmol·L^−1^ NaHCO_3_ solution, with distilled water treatment as the control. Each plant received 50 mL of treatment solution by root irrigation every 3 days. After 2 weeks of treatment, the root systems were carefully removed and thoroughly rinsed with distilled water to remove adhered substrate. Root scanning and analysis were performed using the LA-S root analysis system (Wanshen, Hangzhou, China) [54]. Parameters including total root length, root surface area, root volume, number of root tips, number of root forks, and root projected area were automatically extracted and calculated by the accompanying analysis software.

### 4.8. Alkali Stress Treatment, RNA Extraction, and q-PCR Analysis

CK, GC, GU, and GCU plants were selected as experimental materials. Alkali stress was applied with 100 mmol·L^−1^ NaHCO_3_, with distilled water treatment as the control. Root samples were collected at 0, 3, 6, and 12 h after treatment for RNA extraction and gene expression analysis [17]. Three biological replicates were set for each treatment. Collected root samples were frozen in liquid nitrogen and stored at −80 °C until use. Total RNA was extracted using the TransZol Up Kit (ET111-01-V2; TransGen Biotech Co., Ltd., Beijing, China). First-strand cDNA was synthesized from 1 μg of total RNA using the SPARKSCRIPT II RT Plus Kit (with gDNA Eraser; AG0304-B, Jinan Sparkjade Biotechnology Co., Ltd., Jinan, China) according to the manufacturer’s instructions. Specific primers for *GmCML27*, *GmU2AFb*, and alkali stress-responsive genes are listed in Table 2. q-PCR analysis was performed using SYBR Green Master Mix on a CFX384 Real-Time PCR System (Bio-Rad, Hercules, CA, USA) [55]. The lupine *Ubiquitin* gene was used as the internal reference, and relative expression levels of target genes were calculated using the 2^−^ΔΔCT method.

### 4.9. Data Visualization and Statistical Analysis

Data visualization was performed using GraphPad Prism 10.4.1 software, and schematic diagrams were created with BioGDP (https://BioGDP.com). All data are presented as mean ± standard deviation (SD). Statistical analyses were conducted using GraphPad Prism 10.4.1. Physiological indices, percentage of stained area, root scanning data, and qPCR data were analyzed by two-way ANOVA followed by Tukey’s multiple comparison test. Different lowercase letters indicate significant differences among treatments (*p* < 0.05).

## 5. Conclusions

**Figure 9:**
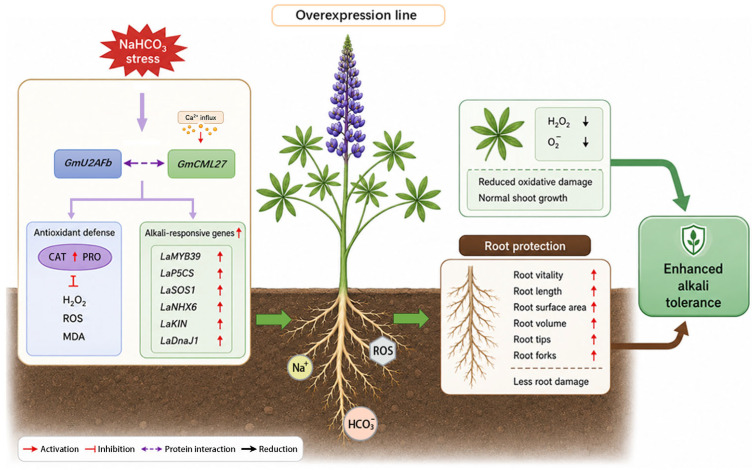
Schematic model of enhanced alkali stress tolerance in lupine via the *GmCML27-GsU2AFb* module. This figure was created using BioGDP (https://BioGDP.com).

In this study, we characterized the potential roles of *GmCML27* and *GmU2AFb* in the alkali stress response of lupine. Bioinformatics analyses revealed that *GmCML27* is a conserved member of the CML family, and preliminary analyses together with protein interaction validation assays supported a potential association between *GmCML27* and *GmU2AFb*. Functional evaluation using an *Agrobacterium rhizogenes*-mediated hairy root transformation system demonstrated that overexpression of either *GmCML27* or *GmU2AFb* enhanced alkali tolerance in lupine, with co-transformed plants exhibiting the strongest tolerance phenotype. Compared with CK plants, transgenic lines maintained higher CAT activity, Pro accumulation, and root vitality, while showing reduced MDA, H_2_O_2_, and ROS accumulation and improved root architecture under alkali stress. Moreover, several alkali stress-responsive genes, including *LaMYB39*, *LaP5CS*, *LaSOS1*, *LaNHX6*, *LaKIN*, and *LaDnaJ1*, showed higher expression levels in transgenic lines. Collectively, these results indicate that *GmCML27* and *GmU2AFb* positively regulate alkali tolerance in lupine and may contribute to stress adaptation through enhanced antioxidant capacity, osmotic regulation, and activation of stress-responsive pathways. This study provides new insights into the functional roles of *GmCML27* and *GmU2AFb* in plant alkali stress responses and establishes a foundation for further investigation of their molecular mechanisms (Figure 9).

## Figures and Tables

**Table 1:** Primers for RT-PCR analysis.

Primer Names	Primer Sequences (5′-3′)
*GmCML2*7-F	ATGGCGACGAATCCAATCG
*GmCML2*7-R	CTAATCGGTTTTTTCTTCGCCA
*GmU2AFb*-F	ATGGCGGAGCACTTGG
*GmU2AFb*-R	CTATATCTGATGGTTACCAGATTC
Ubiquitin-F	GGCAAGACCATCACTCTCGA
Ubiquitin-R	ACCTCAAGGGTGATGGTCT

**Table 2:** Primers used for q-PCR analysis.

Primer Names	Primer Sequences (5′-3′)
*qLaMYB39*-F	TGTCATGGGAAACAAGTGGGC
*qLaMYB39*-R	CGGGGTCAATCCCCATACG
*qLaP5CS*-F	TGTTCTAGACGGCTTCAGGC
*qLaP5CS*-R	AACCCAGCCTAGCAACCAAG
*qLaSOS1*-F	TCTTCACTCTGGCAGGTTCC
*qLaSOS1*-R	CTGTGGGCACGAAGAAATGC
*qLaNHX6*-F	AGGAGCTTGGCACTGATGTC
*qLaNHX6*-R	CAACACCTGCCGACATTGAC
*qLaKIN*-F	ATCTCCATAACGGACTTCGGTG
*qLaKIN*-R	ATTCCCCAACTCCTGCGTGG
*qLaDnaJ1*-F	GGAAATCCATTTGGTGGCGG
*qLaDnaJ1*-R	CCAAGCTGACCTTGAGAGGG
*qUbiquitin*-F	GGCAAGACCATCACTCTCGA
*qUbiquitin*-R	ACCTCAAGGGTGATGGTCT

## Data Availability

The datasets generated during and/or analyzed during the current study are available from the corresponding author on reasonable request.

## Supplementary Materials

The following supporting information can be downloaded at: https://www.mdpi.com/article/10.3390/plants15142196/s1. Figure S1. Predicted aligned error (PAE) analysis of the AlphaFold3-modeled *GmCML27*–*GmU2AFb* complex.
