# Supplementary material for: The Co-Expression of GmCML27 and GmU2AFb Enhances Tolerance to Alkaline Stress in Lupinus angustifolius
Source: Plants (Basel). 2026 Jul 17;15(14):2196. doi: 10.3390/plants15142196 (PMC13417546; doi:10.3390/plants15142196)
Supplement: Supplementary file 1 [file plants-15-02196-s001.zip › plants-4421646-supplementary.pdf]

Supplementary Materials

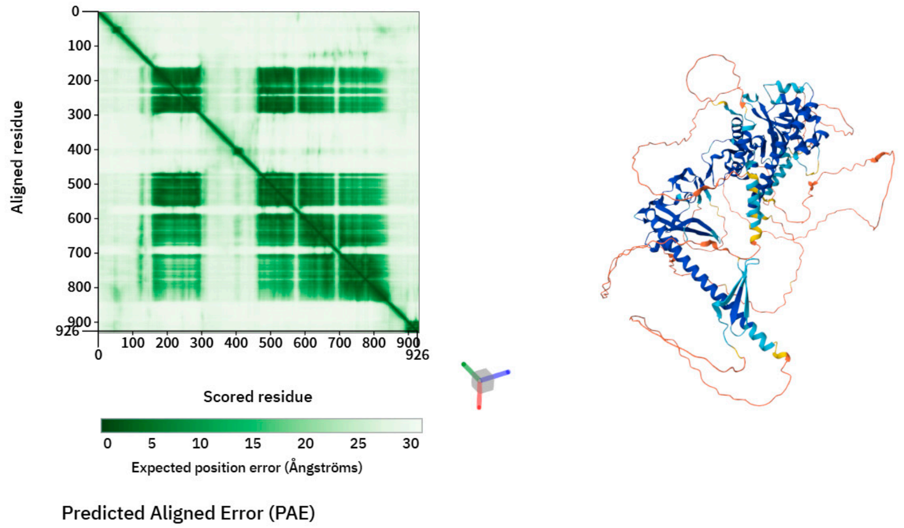

Figure S1. Predicted aligned error (PAE) analysis of the AlphaFold3-modeled *GmCML27–GmU2AFb* complex.
